# Aldehyde dehydrogenase 2 is associated with cognitive functions in patients with Parkinson’s disease

**DOI:** 10.1038/srep30424

**Published:** 2016-07-25

**Authors:** Rwei-Ling Yu, Chun-Hsiang Tan, Ying-Che Lu, Ruey-Meei Wu

**Affiliations:** 1 Institute of Behavioral Medicine, National Cheng Kung University, College of Medicine, Tainan, Taiwan; 2 Department of Psychiatry, National Cheng Kung University Hospital, College of Medicine, National Cheng Kung University, Tainan, Taiwan; 3 Department of Neurology, Kaohsiung Medical University Hospital, Kaohsiung Medical University, Kaohsiung, Taiwan; 4 Ph.D. Program in Translational Medicine, National Taiwan University and Academia Sinica, Taipei, Taiwan; 5 Department of Neurology, National Taiwan University Hospital, College of Medicine, National Taiwan University, Taipei, Taiwan

## Abstract

Neurotransmitter degradation has been proposed to cause the accumulation of neurotoxic metabolites. The metabolism of these metabolites involves aldehyde dehydrogenase 2 (ALDH2). The Asian-specific single nucleotide polymorphism rs671 causes reduced enzyme activity. This study aims to explore whether Parkinson’s disease (PD) patients with reduced ALDH2 activity owing to the rs671 polymorphism are at risk for neuropsychological impairments. A total of 139 PD patients were recruited. Each participant was assessed for medical characteristics and their ALDH2 genotype. The Mini-Mental State Examination (MMSE), the Clinical Dementia Rating Scale and the Frontal Behavioral Inventory were used to measure neuropsychological functions. We found that the MMSE scores were significantly lower in patients with inactive ALDH2 (U = 1873.5, p = 0.02). The presence of cognitive impairments was significantly more frequent in the inactive ALDH2 group (46.0%) than in the active ALDH2 group (26.3%) (χ^2^ = 5.886, p = 0.01). The inactive group showed significant deterioration in hobbies and exhibited more severe “disorganization” and “hyper-sexuality” behaviours. The additive effects of the allele on the development of cognitive impairments in PD patients may be an important finding that provides further insight into the pathogenic mechanism of cognitive dysfunction in PD.

Parkinson’s disease (PD) is a common and progressive neurodegenerative disease that affected 4.1 to 4.6 million people over the age of 50 in 2005, with the numbers expected to double over the next two decades^1^. PD is characterized pathologically by the loss of dopaminergic neurons in the substantia nigra and manifests clinically as resting tremors, rigidity, bradykinesia, and postural instability. Although motor symptoms are the main clinical features of PD, increasing evidence has shown that PD patients also have non-motor symptoms, such as neurocognitive dysfunction and neuropsychiatric symptoms.

Cognitive impairment is one of the most common and devastating non-motor symptoms of PD. One systematic review suggested that the point prevalence of dementia in PD was more than 30%^2^, and individuals with PD had a three-to-six-fold higher risk of developing dementia than people of the same age without PD^3^. Approximately 80–90% of PD patients ultimately develop dementia by the end of longer follow-up periods^4^. Impaired cognitive functioning in PD patients results in a lower quality of life^5^, higher mortality^6^, heavier caregiver burden, and higher health-related costs^7^. Moreover, evidence suggests that the cognitive deficits are more heterogeneous than previously appreciated^8^. Some studies have shown that cognitive dysfunction is most prominent in the domains of memory^9^ and executive function^10^ in PD patients and other studies have shown that impairments in attentional/executive tasks may not always be the predominant deficit in PD^11^. Visuospatial deficits can also sometimes be the most severe cognitive impairments. Some studies have suggested that mild cognitive impairments in PD patients with predominant posterior cortical dysfunctions may result in a more rapid progression to dementia associated with PD (PDD)^12^. These studies clearly show the heterogeneity of neurocognitive dysfunctions in PD patients. However, the reasons for this heterogeneity remain elusive.

Monoamine neurotransmitters including dopamine and norepinephrine are essential components for nervous system functions and have been shown to be important for cognition in numerous studies. The importance of dopamine for cognitive functions is evidenced by the fact that high-level cognitive deficits are common in many dopamine-related disorders, such as addiction^13^ and schizophrenia^14^. The norepinephrine system is also of great importance in cognition based on the results of numerous animal and human studies that have explored the effects of noradrenaline manipulation on attention, working memory, cognitive flexibility, response inhibition and emotional memory^15^. Dopamine and noradrenaline are metabolized by catechol-O-methyl transferase (COMT) or monoamine oxidase (MAO) to intermediate metabolites. If dopamine and noradrenaline are initially metabolized by MAO, the metabolites are 3,4-dihydroxyphenylacetaldehyde (DOPAL) and 3,4-dihydroxyphenylglycolaldehyde (DOPEGAL), respectively. These aldehyde metabolites need to be further metabolized by aldehyde dehydrogenase (ALDH). Growing evidence has suggested that the aldehyde metabolites DOPAL and DOPEGAL are neurotoxic, and their intraneuronal accumulation has been associated with neuronal cell death leading to neurodegeneration^16^,^17^,^18^,^19^,^20^.

There are 19 human aldehyde dehydrogenase isoforms with wide tissue distributions and localizations in all subcellular compartments, including the cytosol, mitochondria, endoplasmic reticulum, and nucleus^21^. DOPAL metabolism primarily occurs in the mitochondrial fraction. Aldehyde dehydrogenase 2 (ALDH2) is a mitochondrial isozyme that has been proposed to be responsible for DOPAL metabolism^22^,^23^,^24^. The metabolism of DOPEGAL by ALDH2 has also been proposed because the DOPEGAL metabolism by ALDH2 is significantly impaired in the presence of acetaldehyde. This finding suggests that these aldehyde metabolites compete with acetaldehyde for ALDH2 metabolism^25^. The ALDH2 single nucleotide polymorphism (SNP) rs671(A), which results in an amino acid change from glutamic acid to lysine at position 504 (Glu504Lys) in the ALDH2 enzyme, is present almost exclusively in Asian populations^26^. The rs671(GG)-encoded enzyme is the active form, whereas the enzyme encoded by the rs671(AG) or rs671(AA) polymorphism is the partially or completely inactive form, respectively, and carries reduced enzymatic activity^27^,^28^. This reduced enzymatic activity has been shown to cause differences in susceptibility to many diseases, such as Asian flush^29^ and a series of cancers^30^.

In view of the crucial role of ALDH2 in the metabolism of the neurotoxic metabolites in the monoamine neurotransmitter pathway and the possible differences in metabolite accumulation caused by the rs671 SNP, we explored whether the rs671 SNP caused differences in the cognitive functions of PD patients.

## Results

A total of 76 patients with rs671(GG) and 63 patients with rs671(AG) and (AA) were included in the study. There were no significant differences in age, gender, education level, age of onset, disease duration, levodopa equivalent daily dose (LED), or disease severity (stage) between the two groups (Table 1).

**Figure 1:**
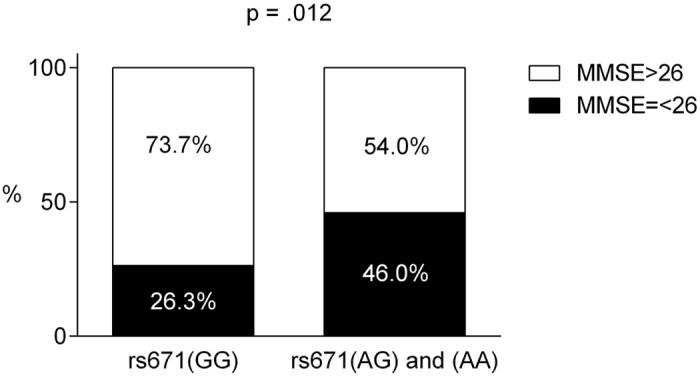
Percentage of patients with MMSE scores higher than or lower/equal to 26 as proposed by the Movement Disorder Society. The presence of an MMSE lower than or equal to 26 was significantly more frequent in the rs671(AG) and (AA) group (46.0%) than the rs671(GG) group (26.3%) (p = 0.012).

In terms of general cognitive functioning, there was a significant difference in the total Mini-Mental State Examination (MMSE) score between the patients with rs671(GG) and rs671(AG) and (AA) (27.24 and 25.73, respectively), with the latter showing a poorer performance (U = 1873.5, p = 0.026). Using the MMSE cut-off value for dementia proposed by the Movement Disorder Society (MDS), we found significant differences between the proportions of patients with dementia in the two groups. The presence of dementia was significantly more frequent in the rs671(AG) and (AA) group (46.0%) than in the rs671(GG) group (26.3%) (χ^2^ = 5.886, p = 0.012). (Fig. 1).

The patients with rs671(GG) tended to score better on all Clinical Dementia Rating Scale (CDR) subscales; however, these differences were only significant for home hobbies (χ^2^ = 7.524, p = 0.023) (Table 2).

**Figure 2:**
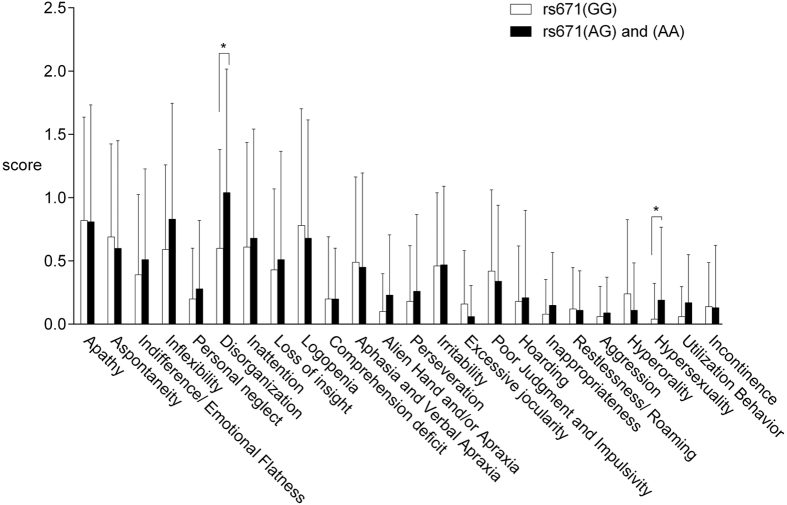
The means and standard deviations of each item on the Frontal behavioral Inventory in the 2 study groups. Patients with rs671(AG) and (AA) showed significantly higher scores for “disorganization” (p = 0.018) and “hyper-sexuality” (p = 0.044).

A total of 98 patients had a caregiver, and these family members completed the Frontal Behavioral Inventory (FBI). All of the caregivers were family members (60% of them were the patients’ spouses, 33% were the patients’ children, and 7% were the patient’s collateral relatives). PD patients with rs671(AG) and (AA) presented more frontal behavioral changes as evaluated by the FBI. When analysing the FBI sub-items, the patients with rs671(AG) and (AA) showed significantly higher scores for “disorganization” (U = 871.5, p = 0.018) and “hyper-sexuality” (U = 1050.0, p = 0.044). However, no significant difference was found in the total FBI scores (Fig. 2).

## Discussion

To the best of our knowledge, no studies have investigated the role of ALDH2 in the cognitive functions of PD patients. We conducted a cross-sectional study to investigate whether there were differences in these neuropsychological functions, including cognitive functioning and behavioural problems, in PD patients carrying different rs671 genotypes. By comparing the enzyme activity between the two study groups, which were satisfactorily matched for demographic and clinical features, we found a significant difference in the total MMSE score between patients with rs671(GG) and rs671(AG) and (AA), with the latter showing a poorer performance. The mean MMSE score of the patients with rs671(AG) and (AA) was 25.7, whereas the mean score of patients with rs671(GG) was 27.2. The MMSE was originally designed to provide a brief, standardized measurement of mental status. The originally recommended MMSE cut-off score of 24 provides good sensitivity for the detection of dementia^31^; however, several recent studies have suggested that this cut-off score may be too low, particularly for PD patients. The MDS Task Force proposed a practical diagnostic procedure^32^ and a diagnostic criteria^33^ for PD dementia in 2007, and an MMSE cut-off score of 26 was advised in the level I testing of the MDS diagnostic procedure. Using this recommended cut-off score, we found a significant difference in the proportions of patients with dementia between the two groups. The presence of dementia was significantly more frequent in the rs671(AG) and (AA) group (46%) than the rs671(GG) group (26%). This result shows that PD patients carrying the rs671(A) allele are at risk for the development of cognitive dysfunctions.

Cognitive impairments amongst PD patients are heterogeneous. Several studies have shown that the cognitive dysfunction of PD patients is most prominent in memory^9^, whereas other studies have shown that the deficit is most prominent in the attentional or executive functions domains^10^,^34^. In the current study, we found that patients with rs671(AG) and (AA) showed significantly worse performances in some MMSE subdomains in addition to the global MMSE score, including attention (i.e., follow a 3-step command) and language function (i.e., make up a sentence). Because the monoamine neurotransmitter system (including dopamine and noradrenaline) is closely related to attention, cognitive flexibility, and response inhibition in PD patients^15^,^35^, we propose that the loss-of-function SNP may cause the degeneration of dopaminergic and noradrenergic neurons through the accumulation of the neurotoxic metabolites (DOPAL and DOPEGAL) of dopamine and noradrenaline. The degeneration of dopaminergic and noradrenergic neurons results in the dysfunction of the attention function and frontal-related behaviour (e.g., disorganization and hyper-sexuality).

The memory function has traditionally been the key criterion for diagnosing dementia; however, growing evidence has shown the heterogeneous nature of cognitive deficits in patients with mild cognitive impairment (MCI) or dementia owing to various aetiologies^36^ (i.e., each cognitive domain plays an equally important role in the diagnosis of dementia). Many diagnostic criteria, such as the DSM-V^37^ and MDS criteria^32^, follow this concept. Several studies have shown that the cognitive profile of dementia in PD is different from that of Alzheimer’s disease (AD) and that this difference exists even in their precursor stages (i.e., the MCI stage). Hence, PD and AD are thought to show different cognitive impairment profiles. Our previous cross-sectional study used comprehensive neuropsychological testing to assess neurocognitive functioning in non-demented PD patients. We found that the cognitive dysfunctions in PD patients predominated in the anterior brain and that executive function was the most vulnerable domain in the cognitive decline process of PD^34^. Previous studies have shown that the ALDH2 SNP may increase the risk of AD development, the core clinical symptom of which is memory dysfunction^38^,^39^. The current study showed that PD patients with reduced ALDH2 activity had worse attention and language functions, which are not the cardinal dysfunction features of AD. This difference in the subdomains of the cognitive impairment profiles compared to typical AD suggests that the mechanisms underlying the cognitive impairments in PD owing to reduced ALDH2 activity are not simply a combination of the deficits of PD and AD. However, the underlying mechanisms for such differences could not be concluded in the current study. Further studies applying comprehensive performance-based neuropsychological test batteries to explore the detailed cognitive profiles caused by the ALDH2 SNP are needed.

In the current study, the severity of dementia symptoms (i.e., its ‘stage’) was quantified by the CDR. No significant difference was found between the two study groups in their global CDR scores, possibly because the CDR assessment tool has been traditionally used to measure the severity of AD-type dementia. Memory is the primary category in the rating process of the CDR’s global score, whereas the other 5 categories are secondary^40^. Thus, we cannot exclude the possibility that the lack of a significant difference between the two study groups may have been due to a lack of sensitivity in the CDR. Although the global CDR scores were similar between the two study groups, we found that the proportion of patients with impairments at home and in hobbies (one sub-domain in the CDR) was significantly different between the 2 groups. A higher percentage of patients carrying rs671(AG) or (AA) (14.3%) had reduced interests and impairments in home functioning than patients carrying rs671(GG) (3.9%). In rating each of these domains, the assessment focused on the patient’s cognitive ability to function in these areas. Limits of the participants in performing activities at home because of motor symptoms, physical frailty, or emotional status do not affect their scoring on the CDR. In other words, only impairments caused by cognitive dysfunction are rated. Therefore, we believe that the reported impairments of daily functioning may be due to the mental decline, such as initiation and organization. Maintaining the household (e.g., laundry, grocery shopping, taking out garbage, or basic home repair) and intellectual interests (e.g., entertaining, painting, or handicrafts) require individual initiation of the motion, planning and arrangement of the affairs day by day. The mental abilities mentioned above are components of executive function^41^, which is the most commonly impaired cognitive domain in PD^34^. Furthermore, when literate caregivers were asked to complete the FBI to measure the patient’s frontal-related behaviours, we found that patients with rs671(AG) and (AA) showed significantly more severe symptoms in their “disorganization” and “hyper-sexuality” behaviours. Disorganization, which is the lack of planning and sequencing, may be one of the earliest behavioural manifestations of the frontal dysexecutive phenomenon^41^,^42^. The findings suggest that ALDH2 inactivation may also result in the development of symptoms related to frontal lobe dysfunction.

ALDH2 is an important enzyme in the metabolism of monoamine neurotransmitters. Individuals carrying the rs671 A allele have reduced enzyme activity, which may lead to inefficient metabolism and accumulation of the neurotoxic metabolites of dopamine and noradrenaline and hence neuronal death and resultant cognitive dysfunction. Because levodopa therapy is one of the key medical treatments, it is possible that the additional dopamine converted from levodopa treatment can lead to increased accumulation of the neurotoxic metabolites and more rapid disease progression. Therefore, it is of great importance to study whether levodopa treatment causes more rapid progression of PD in patients carrying the rs671 A allele.

Although rs671 is an Asian-specific SNP, it is prevalent among Asians and is estimated to be present in more than 540 million individuals worldwide^43^. Recently, we found that cognitive dysfunction was more common in the Asian PD population than in Western PD patients^44^. Determining whether the ethnic difference is partially contributable to this SNP needs further study. However, the importance of ALDH2 in metabolizing the neurotoxic metabolites may not be limited to Asian populations. Genetic variation in ALDH2 among western population was recently shown to exacerbate the PD risk in subjects exposed to ALDH-inhibiting pesticides^24^. These findings suggest that dysregulation of ALDH2 activity through either genetic or environmental factors may be important for pathogenesis in PD patients from different ethnicities.

A potential limitation of the study was the lack of a comprehensive neuropsychological test battery that covered all of the cognitive domains to measure detailed cognitive functioning, particularly executive functioning. Although the MMSE is a well-known tool that is used to assess an individual’s global cognitive functioning, it is used as a screening tool for cognitive impairments. Second, the FBI is a caregiver-report questionnaire and hence may be biased by the responses provided by the caregiver. Therefore, the neurobehavioural problems may be more clearly and objectively evaluated with a semi-structured interview or other objective methods in future studies. Finally, we suggest that future studies should replicate the present findings in an independent PD population sample owing to the small sample size and the possibility of type I error.

In summary, to the best of our knowledge this is the first study to investigate the association between ALDH2 SNP rs671 and neuropsychological functioning in PD patients. The results indicate that PD patients with reduced ALDH2 activity are at risk for the development of cognitive dysfunctions and that these cognitive dysfunctions are different from those associated with AD. PD patients with reduced ALDH2 activity develop neurobehavioural problems significantly more frequently. These findings indicate that the ALDH2 SNP has an important impact on cognitive functioning in PD patients.

## Methods

### Participants

One hundred and thirty-nine patients who were diagnosed with idiopathic PD according to the United Kingdom PD Society Brain Bank clinical diagnostic criteria^45^ were enrolled in this study. The PD patients were recruited via referrals from neurologists at the Movement Disorders Centre. The exclusion criteria included the following: atypical features of parkinsonism, motor symptom onset before 50 years of age, illiteracy, a history of brain operation, and severe systemic disease.

### Standard protocol approvals, registrations, and patient consent

All participants provided written informed consent prior to enrolment in accordance with the ethical standards outlined in the 1964 Declaration of Helsinki. All study procedures were approved by the ethical research committee of National Taiwan University Hospital and Kaohsiung Medical University Hospital. All methods were performed in accordance with the approved guidelines. Each participant underwent a detailed interview to disclose demographic characteristics. Information on dopamine replacement therapy was recorded and the LED was calculated according to the recommendations of Tomlinson *et al.*^46^. Motor severity was quantified using the Hoehn and Yahr (H&Y) staging criteria^47^.

### Neuropsychological assessment

All study participants underwent psychometric testing by a neuropsychologist experienced in the assessment of PD patients. Each patient was assessed while on medication.

General cognitive functioning was measured using the MMSE^31^, which has a maximum score of 30. The MMSE is composed of the following 11 subtests: temporal orientation (5 points), spatial orientation (5 points), immediate memory (3 points), attention/concentration (5 points), delayed recall (3 points), naming (2 points), verbal repetition (1 point), verbal comprehension (3 points), writing (1 point), reading a sentence (1 point), and constructional praxis (1 point).

Dementia severity was assessed using the CDR. The CDR is obtained through semi-structured interviews with patients and informants. Cognitive functioning is rated in the following 6 domains of functioning: memory, orientation, judgement and problem solving, community affairs, home and hobbies, and personal care. Each domain is rated on a 5-point scale of functioning as follows: 0, no impairment; 0.5, questionable impairment; 1, mild impairment; 2, moderate impairment; and 3, severe impairment (personal care is scored on a 4-point scale without a 0.5 rating available). The global CDR score was computed using the Washington University online algorithm^40^. The CDR demonstrates good psychometric properties^48^.

Frontal behavioral changes were measured using the FBI, which rates behavioural changes via caregiver interviews by a trained neuropsychologist using structured questions. The FBI was originally designed to measure behavioural changes in dementia patients and was useful in other disorders affecting frontal systems. The FBI has two subscales for negative and disinhibition behaviours and includes 24 items. Scoring of the answers is based on a 4-point scale of frequency ranging from “none” to “severe or most of the time”, wherein 3 reflects more severe frontal behavioral disorders; the scoring accounts for a total FBI score range of 0 to 72^49^.

### Genotyping

Genetic testing was performed subsequent to the clinical and neuropsychological assessment. Genomic DNA was extracted from peripheral blood leukocytes of the participants with the Genomic DNA Extraction Kit (Geneaid, New Taipei City, Taiwan). ALDH2 rs671(A) genotyping was performed with TaqMan probes and the StepOnePlus™ system with the StepOne software™ (Applied Biosystems, Grand Island, NY, USA). The laboratory technician who performed the genotyping and read the genotype data was blinded to the patients’ clinical data.

### Statistical analysis

Proportions were calculated for qualitative variables and means and standard deviations (SDs) were calculated for quantitative variables. The data were examined for normality and homogeneity of variance. The Chi-square test, t-test, and Mann-Whitney U test were used to examine group differences in nonparametric variables. Because less than 10% of patients had the rs671(AA) genotype, we combined the rs671(AG) and rs671(AA) genotypes for the data analysis. This combination is not illegitimate for the analysis of the acetaldehyde metabolic effects because the rs671(AA)-encoded enzyme is inactive and the rs671(AG)-encoded enzyme has an acetaldehyde metabolite rate that is only one-tenth that of rs671(GG)^50^. Statistical significance was determined when the probability value was less than 0.05. A commercially available software program (SPSS version 17.0; SPSS Inc., Chicago, IL, USA) was used for the statistical analyses.

## Additional Information

**How to cite this article**: Yu, R.-L. *et al.* Aldehyde dehydrogenase 2 is associated with cognitive functions in patients with Parkinson’s disease. *Sci. Rep.* **6**, 30424; doi: 10.1038/srep30424 (2016).

## Figures and Tables

**Table 1:** Demographic and Clinical Characteristics of the Study Groups.

	rs671(GG) (n = 76)		rs671(AG) and (AA) (n = 63)		Statistic	p Value
	mean	SD	mean	SD		
Age (years)	62.99	9.628	64.33	9.125	2216.000	0.451
Gender (female/male)	23/53	—	25/38	—	χ^2^ = 1.352	0.163
Education (years)	11.32	4.431	11.56	4.617	U = 2294.5	0.665
Age at Onset (years)	56.16	9.607	57.43	8.840	U = 2210.0	0.436
Disease duration (years)	6.828	4.605	6.904	6.308	U = 2224.0	0.470
Levodopa equivalent dose	673.413	535.4	641.266	487.333	U = 2253.0	0.755
Hoehn and Yahr stage	2.29	0.995	2.36	1.079	U = 2021.5	0.723
I	23.9%	—	28.8%	—	χ^2^ = 3.069	0.381
II	38.0%	—	23.7%	—		
III	23.9%	—	30.5%	—		
IV	14.1%	—	16.9%	—		

Abbreviations: SD, standard deviation.

**Table 2:** Cognitive functions in the Study Groups.

	rs671(GG) (n = 76)		rs671(AG) and (AA) (n = 63)		Statistic	p Value
	mean	SD	mean	SD		
Mini-Mental State Examination	27.24	3.506	25.73	4.505	U = 1873.5	0.026
Orientation	9.53	0.973	8.97	1.814	U = 2054.5	0.085
Attention	6.99	1.400	6.84	1.461	U = 2260.5	0.540
Memory	2.08	1.030	1.70	1.159	U = 1956.5	0.051
Language	4.86	0.423	4.65	0.600	U = 1993.5	0.014
Naming	2.00	0.000	2.00	0.000	U = 2394.0	1.00
Repetition	100%		100%		—	—
Reading comprehension	96.1%		92.1%		χ^2^ = 1.011	0.261
Make up a sentence	89.5%		73.0%		χ^2^ = 6.325	0.011
Follow a 3-step command	2.93	0.250	2.78	0.552	U = 2128.5	0.042
Construction	81.6%		77.8%		χ^2^ = 0.309	0.364
Clinical Dementia Rating Scale						
0	31.6%		33.3%		χ^2^ = 5.151	0.076
0.5	64.5%		52.4%			
≥1	3.9%		14.3%			
CDR-Memory						
0	34.2%		36.5%		χ^2^ = 2.872	0.238
0.5	47.4%		34.9%			
≥1	18.4%		28.6%			
CDR-Orientation						
0	72.4%		69.8%		χ^2^ = 2.649	0.266
0.5	22.4%		17.5%			
≥1	5.3%		12.7%			
CDR-Judgement						
0	78.9%		66.7%		χ^2^ = 4.271	0.118
0.5	17.1%		20.6%			
≥1	3.9%		12.7%			
CDR-Community Affairs						
0	77.6%		76.2%		χ^2^ = 1.941	0.379
0.5	17.1%		12.7%			
≥1	5.3%		11.1%			
CDR-Home Hobbies						
0	78.9%		79.4%		χ^2^ = 7.524	0.023
0.5	17.1%		6.3%			
≥1	3.9%		14.3%			
CDR-Personal Care						
0	93.4%		85.7%		χ^2^ = 2.259	0.111
≥1	6.6%		14.3%			
